# Is pre-breeding prospecting behaviour affected by snow cover in the irruptive snowy owl? A test using state-space modelling and environmental data annotated via Movebank

**DOI:** 10.1186/s40462-015-0028-7

**Published:** 2015-01-17

**Authors:** Jean-François Therrien, David Pinaud, Gilles Gauthier, Nicolas Lecomte, Keith L Bildstein, Joël Bety

**Affiliations:** Hawk Mountain Sanctuary, Acopian Center for Conservation Learning, Orwigsburg, PA 17961 USA; CEBC, UMR7372, CNRS/Univ La Rochelle, 79360 Villiers en Bois, La Rochelle, France; Département de Biologie & Centre d’Études Nordiques, Université Laval, Québec, G1V 0A6 Canada; Canada Research Chair in Polar and Boreal Ecology, Université de Moncton, Moncton, E1A 3E9 Canada; Département de Biologie & Centre d’Études Nordiques, Université du Québec à Rimouski, Québec, G5L 3A1 Canada

**Keywords:** Dispersal, Env-DATA, Movebank, Pre-breeding movements, Snow, Snowy owl, State-space model

## Abstract

**Background:**

Tracking individual animals using satellite telemetry has improved our understanding of animal movements considerably. Nonetheless, thorough statistical treatment of Argos datasets is often jeopardized by their coarse temporal resolution. State-space modelling can circumvent some of the inherent limitations of Argos datasets, such as the limited temporal resolution of locations and the lack of information pertaining to the behavioural state of the tracked individuals at each location. We coupled state-space modelling with environmental characterisation of modelled locations on a 3-year Argos dataset of 9 breeding snowy owls to assess whether searching behaviour for breeding sites was affected by snow cover and depth in an arctic predator that shows a lack of breeding site fidelity.

**Results:**

The state-space modelling approach allowed the discrimination of two behavioural states (searching and moving) during pre-breeding movements. Tracked snowy owls constantly switched from moving to searching behaviour during pre-breeding movements from mid-March to early June. Searching events were more likely where snow cover and depth was low. This suggests that snowy owls adapt their searching effort to environmental conditions encountered along their path.

**Conclusions:**

This modelling technique increases our understanding of movement ecology and behavioural decisions of individual animals both locally and globally according to environmental variables.

## Background

Tracking individual animals using satellite telemetry has improved our understanding of their movement ecology considerably by providing databases that otherwise would be impossible to obtain. Indeed, the last several decades have nurtured a rise in the number of studies using the Argos system to analyse the movements of individual animals through time [1]. However, even with those ever-growing datasets of animal locations, we are still limited in our ability to address some broad questions pertaining to movement ecology of organisms. An important limiting factor for understanding the functional hierarchy of decisions underlying movements of individuals is our narrow set of analytical tools that can couple raw locations of individuals with any of the four basic mechanistic components of movement (i.e. motion and navigation capabilities as well as internal and external states), as described by Nathan et al. [2].

Indeed, thorough statistical treatment of Argos datasets and interpretation of specific animal behaviour during movement are hampered by three major limitations. First, those datasets have a coarse temporal resolution and often have irregular location estimates because most transmitters use programmed duty cycles and are dependent on the communication strength with moving satellites. This temporal irregularity increases the complexity of the data and prevents the use of common statistical analyses. Second, the locations themselves only come with a quality indicator, derived from the number of signals received, but do not provide clues as to what behaviour the individual animal was performing other than where it was. Third, the difficulty in linking any set of environmental conditions that are heterogeneous in source and format, and often obtained at different spatiotemporal scales than movement data, with any set of locations of an individual, often prevent users of answering rather basic questions as to what is affecting movements of the tracked organisms (but see [3,4] for examples).

State-space hierarchical switching modelling (hSSSM; [5]) can circumvent some of the inherent limitations of a typical Argos dataset, such as the restricted temporal resolution of locations and the lack of information pertaining to the behavioural state of the tracked individuals at each location. Indeed, this Bayesian approach allows the estimation of the most probable locations at fixed time steps based on the previous and forthcoming locations while taking into account the accuracy of each location provided by CLS Argos (see [5,6] for further details; CLS stands for Collecte Localisation Satellite). One can thus estimate locations at regular time steps and use them to perform subsequent movement analyses. Moreover, for each estimated location, the model can assess the probability of an individual being in one of two (or more, see [5]) predefined contrasting behavioural states according to proxies such as travel speed and turn angles, providing useful behavioural classifications (see [7] for a review). Such behavioural assessment can improve our understanding of underlying processes occurring at the individual level and, ultimately, of movement ecology. In addition, the recent development of the Environmental-Data Automated Track Annotation System of Movebank (Env-DATA; [8]) now allows automatic annotation of movement trajectories with instantaneous environmental conditions using large volumes of environmental data. This publicly available system eliminates the technical difficulties related to the annotation process such as data acquisition and interpolation, as described by Dodge et al. [8]. By coupling those two promising techniques (SSM and annotation of locations), we here show how movements of animals tracked with the Argos system can be influenced by environmental conditions encountered.

As one of the main predators of the tundra [9,10], the snowy owl (*Bubo scandiacus*) shows some of the most spectacular irruptive movements in boreal areas [11,12], concentrating in high density at some sites in given years and deserting them completely in subsequent years. Recent tracking studies were able to document breeding dispersal at the individual level and reported an almost complete lack of fidelity to breeding sites [11,12]. Those movements have been correlated with the abundance of lemmings (*Lemmus* and *Dicrostonyx* sp.; [13,14]) since snowy owls feed almost exclusively (>95%) on those small mammals during summer and rely on them for reproduction [10,12,15]. Those small rodents show high-amplitude variations in abundance across years at a given site [16,17]. Such fluctuations can be spatially unpredictable [18,19], triggering long-distance movement of owls between breeding attempts to find sites harbouring high lemming densities [12]. While prospecting during the pre-breeding period (March - May), owls have been reported to reduce their speed and increase their turn angles in certain areas with more directional movements in between these areas [12]. The zigzagging movements shown by owls in some areas during prospecting are likely used to assess nesting conditions, and especially whether lemming density is high enough to entice owls to settle in that area for breeding. This prospecting occurs when the ground is still almost 100% snow-covered and the precise mechanisms used by owls to assess lemming densities remain unknown but likely include auditory and/or visual cues [14].

We hypothesized that pre-breeding prospecting movements of snowy owls are affected by environmental conditions encountered during this period. For instance, a thick snow cover may prevent owls from hearing or seeing lemmings or their signs (e.g. tracks) or denuded sites at the margin of snow banks can facilitate lemming catching by owls. In addition, snowy owls are facing a time constraint during the pre-breeding season and individuals should benefit from finding a suitable breeding site as early as possible. Indeed, competition for territories is likely high as nesting sites with good food resources are probably limited. Moreover, an earlier onset of breeding is likely to increase reproductive success in this harsh environment characterized by a short summer, as observed in many other Arctic species [20,21]. Therefore, we predicted that snowy owls should spend less time searching in areas with a thick or extended snow cover, and we expected owls to settle where snow cover and depth are lower than average. Here we show how the combination of SSM with the Env-DATA System can help addressing this hypothesis.

## Results

We tracked 9 of the 12 marked female snowy owls during the first year and 7 of them for an additional 2 years. Over the years, tracked snowy owls began their pre-breeding prospecting movements on (± SD) 5 April ± 17 days and continued to do so for 36 ± 25 days. During that time, birds covered 1251 ± 1175 km. The birds often alternated between the moving and searching states, patrolling several distinct searching areas before eventually settling for the summer. Owls actually settled for summer on 12 May ± 21 days.

**Figure 1:**
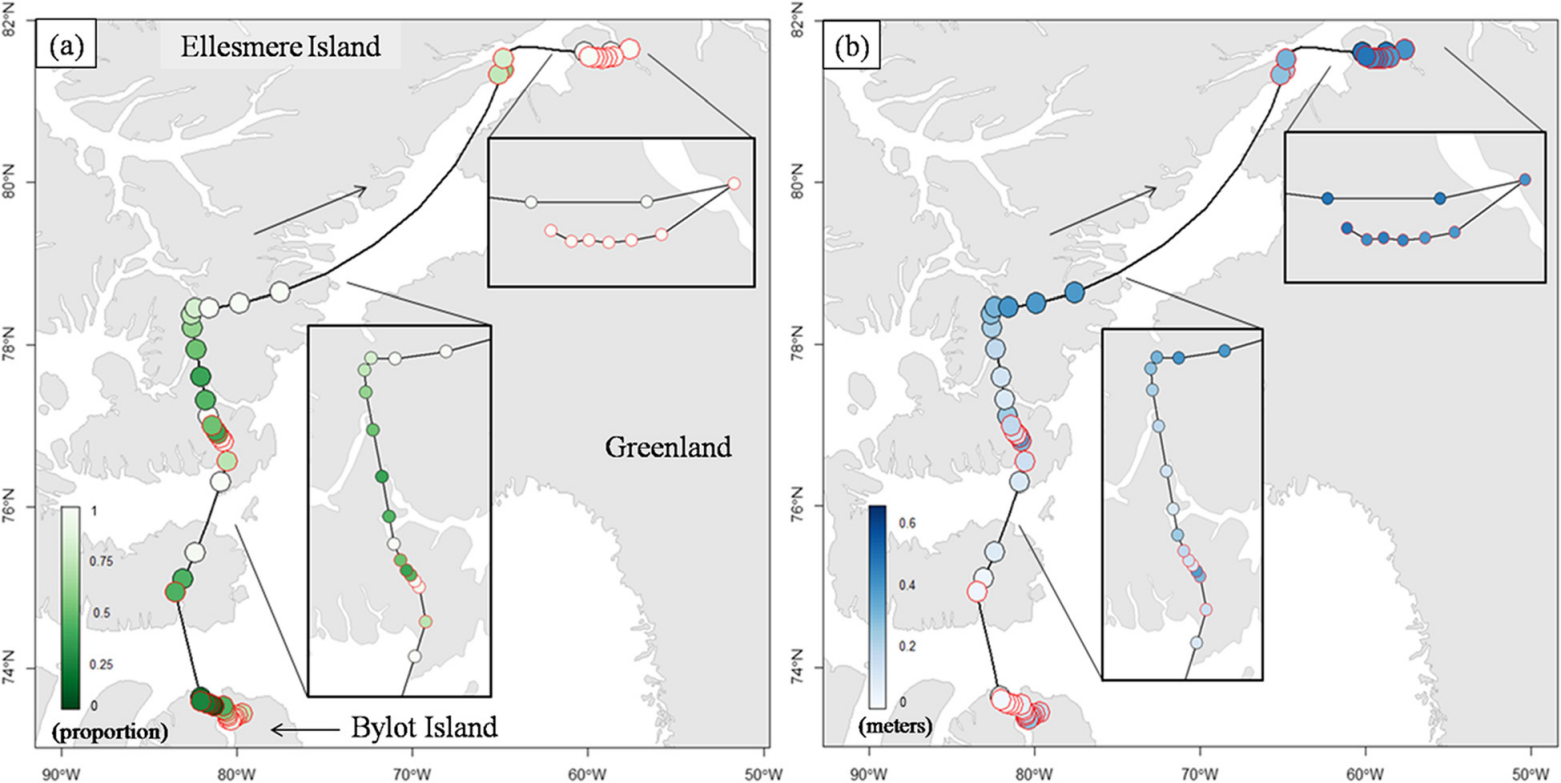
**Example of snowy owl’s pre-breeding movements, alternating between searching and moving behavioural states.** Example of a tracked snowy owl searching for a potential breeding site in the Arctic in spring 2009, in relation with surface snow cover **(a)** and depth **(b)**. Red-outlined dots indicate locations where searching behavioural state occurred.

We used a total of 181 moving and 480 searching locations on land in the analyses (Figure 1). Average snow cover encountered by the tracked birds varied significantly over time throughout the pre-breeding prospecting period in only one year (2009: F_1,261_ = 25.7, P < 0.01) out of three (2008: F_1,233_ = 0.02, P = 0.9; 2010: F_1,167_ = 2.2, P = 0.14). The decrease in snow cover in that year (2009) was negligible over the prospecting period (−0.33% per day or 12% over the whole period). The overall average snow cover encountered by owls during all years was 78 ± 36%. Average snow depth however showed light but significant downward trends over time in all three years of tracking (2008: F_1,233_ = 17.5, P < 0.01; 2009: F_1,261_ = 20.5, P < 0.01; 2010: F_1,167_ = 22.0, P < 0.01), representing a decrease of 0.09, 0.05 and 0.11 m over the prospecting period in 2008, 2009 and 2010 respectively. The overall average snow depth encountered by owls during all years was 0.27 ± 0.21 m.

**Figure 2:**
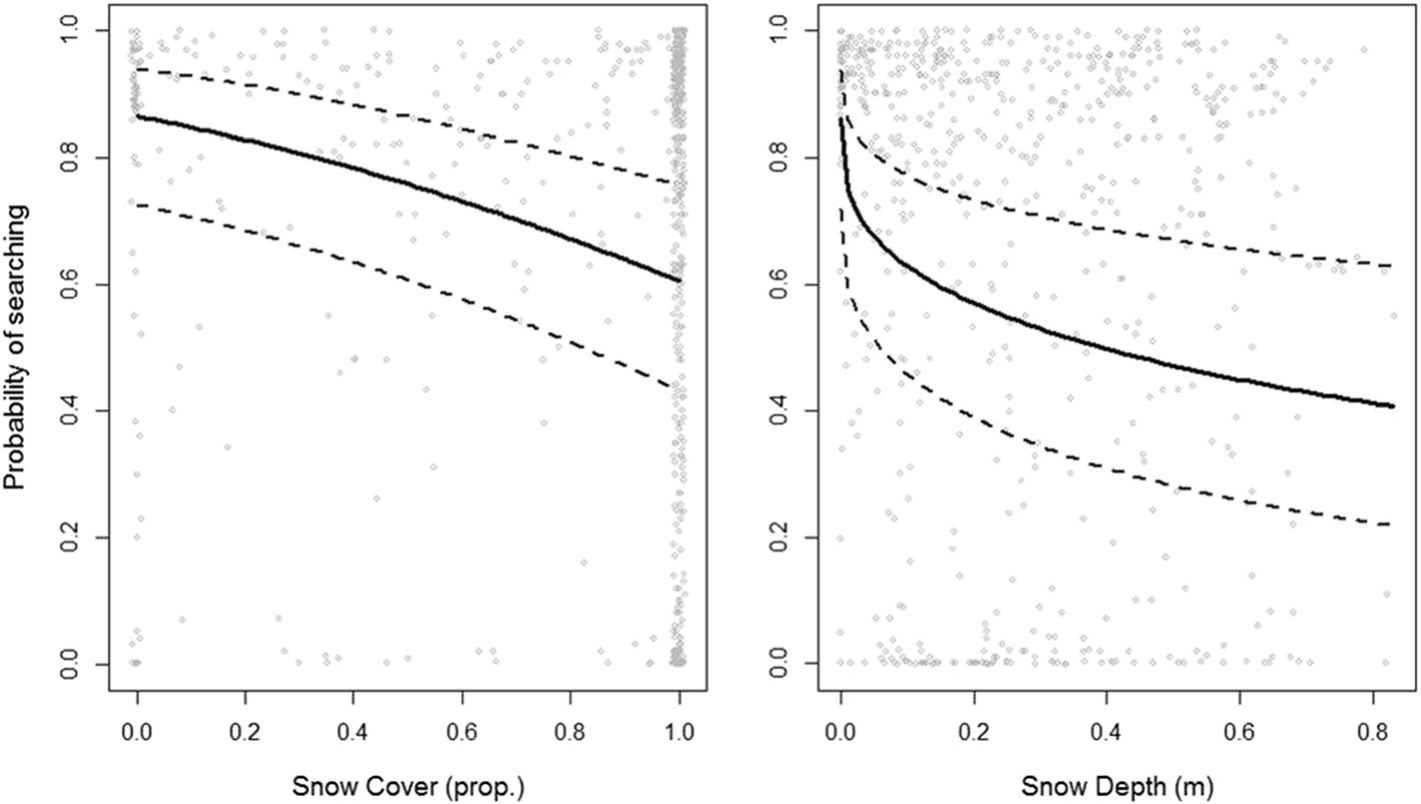
**Searching probability of snowy owls during pre-breeding movements in relation to snow cover and depth.** Probability of entering into a searching behavioural state during pre-breeding prospecting movements as a function of snow cover and depth in 9 snowy owls tracked via satellites in the Arctic in 2008–2010. For each panel, the predicted probabilities from the final mixed model is shown (calculated at the population level) with 95% confidence intervals.

**Figure 3:**
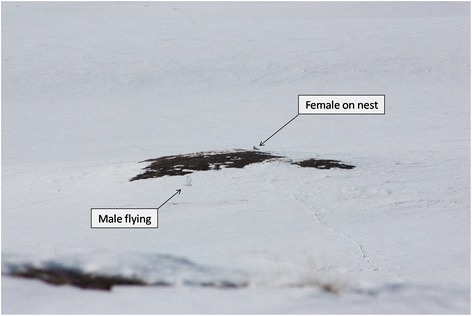
**Breeding snowy owl pair at the onset of nesting, Canada, 23 May 2014.** A snowy owl breeding pair at the onset of nesting, showing the extent of snow cover typical at that time of year. Photo credits: C. Chevalier, Bylot Island, Canada, 23 May 2014.

The probability of being in a searching state was not constant, as we found a significant negative relationship between searching probability and both snow cover (slope ± SE = −1.43 ± 0.37, variance explained = 20.5%) and depth (slope ± SE = −2.27 ± 0.60, variance explained = 20.5%; Table 1, Figure 2). Those covariates were however highly correlated (R = 0.65, P < 0.01, df = 659) and we therefore tested their effect on searching probability separately in 2 different models. Across the range of snow cover encountered by owls during their prospecting movements, the probability of being in searching state increased from 0.60 to 0.87 when birds passed from an area with 100% of snow cover to 0%. Similarly, the probability of being in searching state decreased from 0.86 to 0.41 when birds passed from an area with no snow to 0.80 m of snow. The average snow cover and depth (± SD) when owls settled at their breeding site was 69 ± 42% and 0.23 ± 0.18 m, respectively (Figure 3).

**Table 1:** **Results of model selection for the effect of two snow variables on the probability that 9 snowy owls entered into a searching behavioural state during their pre-breeding prospecting movements in northern Canada in 2008, 2009 and 2010**

**Variable tested**	**Model**	**df**	**ΔAIC**	**logLik**	**Deviance**
snow cover	snow cover	5	0	−1671	3343
	null	4	12.5	−1679	3357
snow depth	snow depth	5	0	−347.3	694.7
	null	4	6.0	−351.3	702.7

In all models, individual’s ID and year (nested in animal ID) were included as random factors (n = 661 locations).

## Discussion

Our study showed that in snowy owls, there is a strong relation between prospecting behaviour (switching from moving to searching state) during pre-breeding movements and environmental conditions encountered and more specifically snow cover and depth. Indeed, birds tended to pass quickly and follow a direct path over areas with more complete or thicker snow cover and were more prone to enter into searching behavioural state when encountering a thinner or reduced snow cover. Although the owls’ diet during the pre-breeding period is not known precisely, lemmings are likely to be the dominant prey during that time, as it is the case during the breeding period, and snow cover can be a refuge for small mammals against avian predators. Indeed, hunting success was reduced by snow depth in wintering snowy owls feeding on small mammals [22]. A thick snow cover is therefore likely to reduce snowy owls’ ability to detect signs of lemming presence as well as their capacity to actually catch them. In order to maximise resource acquisition early during the breeding season, owls may thus increase their searching behaviour in areas where their capacity to find and catch prey is high.

Reproduction in snowy owls is tightly linked with lemmings [10] and those cyclic populations vary tremendously in abundance from one year to another at a given location. Their reproductive success is thus driven by their capacity to cope with the spatial unpredictability of food sources when finding a suitable nesting site. Indeed, most sites within the breeding range of snowy owls are actually vacated when lemming numbers are low (e.g. [9,12,14]) and only transient or non-breeding birds are occasionally observed in those years ([23], N. Lecomte unpubl. data; F. Doyle pers. comm.). These highly mobile predators are thus covering hundreds of kilometres during the short time window of pre-breeding movements to find a site harbouring a high density of lemmings [12]. Finding a good nesting territory as early as possible likely increases their reproductive success by ensuring access to a high abundance of food to raise their chicks and allowing young to reach the critical period of independence not too late during the summer, before the onset of the harsh winter conditions. Patrolling over a site with a thick snow cover in spring should hamper their ability or increase the time required to accurately assess lemming abundance. Therefore, it makes sense for owls to concentrate their searching behaviour in areas where this assessment is easier and quicker, such as areas with little snow, considering the potentially high cost of making a poor or a late decision in their settlement.

It could be argued that in snowy owls, the male is the sex selecting a nesting site and therefore should display such searching behaviour [14]. In our study, tracked females nonetheless exhibited extended prospecting behaviour every year, as described elsewhere [11,12]. This offers a new perspective on how pre-breeding strategies are expressed between sexes in this species. Females are likely building up their body reserves as well as looking for an optimal site for egg-laying, which can be more easily achieved where snow cover is thin or absent. If males adopt a similar strategy during the pre-breeding period and also concentrate their activity in those areas, this could also increase the chances of having a successful *rendezvous* when looking for a mate. Moreover, the possibility that the two sexes are travelling together during this period remains to be studied. Whether searching behaviour of males and females differ in terms of timing and intensity remains an open question and requires males equipped with transmitters during the pre-breeding season.

Even though owls concentrated their searching behaviour in areas with a relatively low and thin snow cover, they eventually settled in areas with moderate snow cover and depth (around 70% of cover and 0.25 m of depth) and not in areas with little snow. In winter, lemmings concentrate in areas of extensive and especially deep snow cover [24,25], a good winter habitat for them where their survival and perhaps even reproduction is high [26]. Therefore, owls may be facing a trade-off when they settle as areas with more snow have the potential to harbour a higher density of lemmings but the quality of these sites may be more difficult to assess and prey may be more difficult to access for a longer period of time, i.e. until snow-melt.

Snow cover and depth will be affected by the expected environmental changes occurring presently in the Arctic and over the next decades. Recent climate models predict reduction in snow cover and increased snow thinning in most arctic regions in spring, and such a reduction has already been documented in northern Canada [27]. A thinner and sparser snow cover may facilitate lemming detection by owls, but it might also reduce winter habitat quality for lemmings, thus reducing their overall availability. Other issues, such as increasing rain-on-snow events creating crusty snow that prevent herbivores from reaching their food under the snow [28,29] may also negatively affect accessibility to prey for predators like owls. Such events are likely to affect the prospecting behaviour of owls and they may need to increase the spatial scale of their searching movements.

## Conclusions

The use of state-space hierarchical switching modelling as described by Jonsen et al. [5] allowed us to perform basic movement analyses on locations regularly spaced in time even though the raw Argos data were not. Moreover, this approach allowed us to incorporate estimates of uncertainty around raw Argos locations, while simultaneously providing us with parameter-based estimates of behaviour. With this approach, we were able to extract detailed information pertaining to the behavioural state of the tracked individuals. By doing so, we categorized critical behaviour (searching and moving) that related to key life cycle events (pre-breeding prospecting and summer settlement) of snowy owls. Behavioural discrimination using state-space hierarchical switching models has been used in the past in totally different species and environments (see [5,30,31]) for examples with seals, turtles and thrushes respectively). This versatile approach is however likely to be limited in the number of different behavioural states that it can successfully discriminate by the accuracy and temporal resolution of any dataset [3,30]. Finally, by combining this method with the annotation of environmental variables using the Env-DATA System of Movebank [8], we merged two modelling approaches to generate both detailed maps and relevant biological relationships. We here provided detailed evidence of how an avian predator of the tundra adapts its behaviour according to the conditions encountered during its pre-breeding prospecting movements. This coupling of techniques opens the possibility of using predicted variations in snow fall due to climate change to generate predictive maps of future sensitivity to snow attributes for a key arctic predator, early in the season. This approach thus increases our understanding of movement ecology and behavioural decisions of individual animals both locally and globally according to environmental variables.

## Methods

Field activities took place on the southern portion of Bylot Island, Nunavut, Canada (73°N, 80°W). From 27 June to 11 July 2007, we captured 12 adult female snowy owls on their nests using a bow-net trap. We equipped owls with 30-g satellite transmitters (Microwave Telemetry, MD, USA; PTT-100) attached as a back-pack with a harness made of Teflon ribbon (see [32] for details). Tracking these birds for up to 3 years suggested no impact of the transmitter on their subsequent survival or reproduction [32]. During the first spring and summer (March to July 2008), transmitters were programmed to transmit for 5 h and then turn off for 49 h. Cycles of 4 h of transmission and 142 h off were programmed for the remaining battery life of the transmitters, including subsequent spring migration. Since we were interested in movement patterns on a broad temporal scale, those variable settings were programmed to increase transmitter life expectancy. We received locations of marked owls via the Argos system [6]. Each location was assigned by CLS Argos to a class (0, 1, 2, 3, A, B, or Z) according to its estimated accuracy, using the least squares filtering process. The accuracy of location classes 0, 1, 2, and 3 are > 1000 m, < 1000 m, < 350 m, and < 150 m, respectively [6]. There is no accuracy estimate associated with the remaining classes (A, B, and Z). From 1 March to 30 June, we received an average of 440 (range: 347–546), 161 (range: 121–226), and 135 (range: 98–216) locations per bird from CLS Argos in 2008, 2009, and 2010, respectively. Overall, 9%, 19%, 29%, 29%, 6%, 7%, and 1% of locations were of quality 3, 2, 1, 0, A, B, and Z, respectively.

We applied a state-space hierarchical switching model (hSSSM, [5]) to estimate daily locations of individual birds during pre-breeding prospecting movements for each year of monitoring (n = 3), using almost all locations (see below) provided by CLS Argos (see [12] for details). For each location, hSSSM assigns the probability that a bird was in a moving (probability close to 0) or searching (probability close to 1) behavioural state according to its speed and turning angle. hSSSM estimations were made using the R package bsam [33] under the R 2.15.2 environment (R Core Team) with the Markov Chain Monte Carlo (MCMC) sampler of JAGs [34]. Basic movement analyses were done using the trip package [35]. To ensure accurate and faster estimates with the hSSSM, we applied a speed filter (500 km/h, [36]) before running estimations to remove extreme error locations. The state-space hierarchical switching models were fitted to the dataset using 2 chains of 250 000 MCMC samples; the first 100 000 samples were discarded as a burn-in and the remaining 150 000 were thinned out to 3000 samples by retaining only every 50th sample to reduce autocorrelation. Estimations of parameters were based on these final 3000 samples. For each estimated parameter, convergence and absence of autocorrelation were checked, and we also applied the convergence diagnostic of Gelman and Rubin [37].

We defined pre-breeding prospecting movements as those occurring between the first time a bird switched to a searching state over potential breeding habitat after departing from their wintering grounds and the time of settlement for the summer. We defined individual departure date from wintering sites when behaviour state switched for the first time from searching to moving after 1 March (see [12] for details). A bird was deemed to have settled on a potential breeding site when it entered into continuous searching state and stayed in a 5-km radius restricted area (i.e. home range) throughout July ([38]; see also [12,33] for details on how summer settlement and home range were calculated).

Locations estimated with hSSSM were annotated with snow cover (%) and depth (m) at surface for the date of each location from the North American Regional Reanalysis of the National Center for Environmental Prediction (NCEP NARR). Original data were provided by the Physical Science Division of the US National Oceanic and Atmospheric Administration Earth System Research Laboratory. The datasets have a spatial and temporal granularity of 0.3 degrees and 20 minutes, respectively. We used the bilinear interpolation method through the Env-DATA System of Movebank [8].

We compared the snow cover and depth encountered during moving and searching behaviour states to examine behavioural responses to variations in environmental conditions experienced along the track [39]. As multiple dependencies were present in the dataset (measure of searching probabilities at different locations by the same individuals through several years), a general linear mixed models approach was used. For each model, residuals were inspected for linearity and homoscedasticity assumptions [40], and a transformation of the explanatory variable was applied when relevant. Models were fitted using R 3.1.1 (R core team 2014) and general linear mixed models (lme4 R package, [41]), with individual’s ID and year (nested in animal ID) as random factors and the response variable (searching probability) was logit-transformed. As nest sites and main prey (lemmings) occur exclusively on land, we limited our analyses to locations over land even though owls can travel over the sea during their annual movements [42]. We also evaluated snow cover and depth at the date that owls actually settled at a site to breed. The significance of models with either of the two variables of interest (snow cover and depth, this latter variable was transformed by squaring to reach model assumptions) was assessed using the information theoretic approach based on AIC [43] and comparison to a null model [40].

## References

[1] Douglas DC, Weinzierl R, Davidson SC, Kays R, Wikelski M, Bohrer G (2012). Moderating Argos location errors in animal tracking data. Meth Ecol Evol..

[2] Nathan R, Getzb WM, Revillac E, Holyoak M, Kadmona R, Saltze D, Smousef PE (2008). A movement ecology paradigm for unifying organismal movement research. PNAS..

[3] Lowther AD, Harcourt RG, Page B, Goldsworthy SD (2013). Steady as he goes: at-sea movement of adult male Australian sea lions in a dynamic marine environment. PLoS One..

[4] Blanchet MA, Lydersen C, Ims RA, Lowther AD, Kovacs KM (2014). Harbour seal *Phoca vitulina* movement patterns in the high-Arctic archipelago of Svalbard, Norway. Aquat Biol..

[5] Jonsen ID, Flemming JM, Myers RA (2005). Robust state-space modeling of animal movement data. Ecology..

[6] Collecte Localisation Satellites (2011). Argos User ’s Manual.

[7] Hawkes C (2009). Linking movement behaviour, dispersal and population processes: is individual variation a key?. J Anim Ecol..

[8] Dodge S, Bohrer G, Weinzierl R, Davidson SC, Kays R, Douglas D, Cruz S, Han J, Brandes D, Wikelski M (2013). The environmental-data automated track annotation (Env-DATA) system: linking animal tracks with environmental data. Movement Ecology..

[9] Gilg O, Sittler B, Sabard B, Hurstel A, Sane R, Delattre P, Hanski L (2006). Functional and numerical responses of four lemming predators in high arctic Greenland. Oikos..

[10] Therrien JF, Gauthier G, Korpimaki E, Bêty J (2014). Predation pressure by avian predators suggests summer limitation of small mammal populations in the Canadian Arctic. Ecology..

[11] Fuller M, Holt D, Schueck L, Berthold P, Gwinner E, Sonnenschein E (2003). 2003 Snowy Owl Movements: Variation on the Migration Theme. Avian Migration.

[12] Therrien JF, Gauthier G, Pinaud D, Bêty J (2014). Irruptive movements and breeding dispersal of snowy owls: a specialized predator exploiting a pulsed resource. J Avian Biol..

[13] Newton I (2006). Advances in the study of irruptive migration. Ardea..

[14] Parmelee DF, Poole A, Stettenheim P, Gill F (1992). Snowy Owl. The Birds of North America.

[15] Therrien JF, Gauthier G, Robillard A, Lecomte N, Bêty J: Écologie de la reproduction du harfang des neiges dans l’Arctique canadien. Naturaliste Canadien. 2015;139:17-23. In French, with English abstract.

[16] Ims RA, Fuglei E (2005). Trophic interaction cycles in tundra ecosystems and the impact of climate change. Bioscience..

[17] Krebs CJ (2011). Of lemmings and snowshoe hares: the ecology of northern Canada. Proc R Soc B..

[18] Myrberget S (1973). Geographical synchronism of cycles of small rodents in Norway. Oikos..

[19] Ims RA, Andreassen HP (2000). Spatial synchronization of vole population dynamics by predatory birds. Nature..

[20] Lepage D, Gauthier G, Menu S (2000). Reproductive consequences of egg-laying decisions in snow geese. J Anim Ecol..

[21] Perrins CM (1970). The timing of birds’ breeding seasons. Ibis..

[22] Chamberlin ML (1980). Winter hunting behavior of a snowy owl in Michigan. Wilson Bull..

[23] Pitelka FA, Tomich PQ, Treichel GW (1955). Ecological relations of jaegers and owls as lemming predators near Barrow, Alaska. Ecol Monogr..

[24] Duchesne D, Gauthier G, Berteaux D (2011). Habitat selection, reproduction and predation of wintering lemmings in the Arctic. Oecologia..

[25] Reid D, Bilodeau F, Krebs CJ, Gauthier G, Kenney AJ, Gilbert BS, Leung MCY, Duchesne D, Hofer E (2012). Lemming winter habitat choice: a snow-fencing experiment. Oecologia..

[26] Bilodeau F, Gauthier G, Berteaux D (2013). The effect of snow cover on lemming population cycles in the Canadian High Arctic. Oecologia..

[27] Derksen C, Brown R (2012). Spring snow cover extent reductions in the 2008–2012 period exceeding climate model projections. Geophys Res Lett..

[28] Hansen BB, Aanes R, Herfindal I, Kohler J, Saether BE (2011). Climate, icing, and wild arctic reindeer: past relationships and future prospects. Ecology..

[29] Stien A, Ims RA, Albon SD, Fuglei E, Irvine RJ, Ropstad E, Halvorsen O, Langvatn R, Loe LE, Veiberg V, Yoccoz NG (2012). Congruent responses to weather variability in high arctic herbivores. Biol Letters..

[30] Jonsen ID, Myers RA, James MC (2007). Identifying leatherback turtle foraging behaviour from satellite telemetry using a switching state-space model. Mar Ecol Prog Ser..

[31] Vergara PM, Pérez-Hernández CG, Hahn IJ, Jiménez JE (2013). Matrix composition and corridor function for austral thrushes in a fragmented temperate forest. Landsc Ecol..

[32] Therrien JF, Gauthier G, Bêty J (2012). Survival and reproduction of adult snowy owls tracked by satellite. J Wildl Manage..

[33] Jonsen ID, Basson M, Bestley S, Bravington MV, Patterson TA, Pedersen MW, Thomson R, Thygesen UH, Wotherspoon SJ (2013). State-space models for bio-loggers: a methodological road map. Deep-Sea Res II..

[34] Plummer M: rjags: Bayesian graphical models using MCMC. R package version 3–9, http://CRAN.R-project.org/package=rjags; 2012.

[35] Sumner MD: trip: spatial analysis of animal track data. R package version 1.1-12, http://CRAN.R-project.org/package=trip; 2012.

[36] McConnell BJ, Chambers C, Fedak MA (1992). Foraging ecology of southern elephant seals in relation to the bathymetry and productivity of the Southern Ocean. Antarct Sci..

[37] Gelman A, Rubin DB (1992). Inference from iterative simulation using multiple sequences. Stat Science..

[38] Ganusevich SA, Maechtle TL, Seegar WS, Yates MA, McGrady MJ, Fuller M, Schueck L, Dayton J, Henny CJ (2004). Autumn migration and wintering areas of Peregrine Falcons *Falco peregrinus* nesting on the Kola Peninsula, northern Russia. Ibis..

[39] Kotliar NB, Wiens JA (1990). Multiple scales of patchiness and patch structure: a hierarchical framework for the study of heterogeneity. Oikos..

[40] Zuur AF, Ieno EN, Walker NJ, Saveliev AA, Smith GM (2008). Mixed Effects Models and Extensions in Ecology With R.

[41] Bates DM, Maechler M, Bolker B, Walker S, Christensen RHB, Singmann H, Dai B: lme4: Linear mixed-effects models using Eigen and S4. R package version 1.1-7, http://cran.r-project.org/web/packages/lme4/; 2014.

[42] Therrien JF, Gauthier G, Bêty J (2011). An avian terrestrial predator of the Arctic relies on the marine ecosystem during winter. J Avian Biol..

[43] Burnham KP, Anderson DR (2002). Model Selection and Multimodel Inference: A Practical Information-Theoretical Approach.

